# Multimorbidity: Epidemiology and Risk Factors in the Golestan Cohort Study, Iran: A Cross-Sectional Analysis

**DOI:** 10.1097/MD.0000000000002756

**Published:** 2016-02-18

**Authors:** Batoul Ahmadi, Masoomeh Alimohammadian, Mehdi Yaseri, Azam Majidi, Majid Boreiri, Farhad Islami, Hossein Poustchi, Mohammad H. Derakhshan, Akabar Feizesani, Akram Pourshams, Christian C. Abnet, Paul Brennan, Sanford M. Dawsey, Farin Kamangar, Paolo Boffetta, Alireza Sadjadi, Reza Malekzadeh

**Affiliations:** From the Department of Health Management and Economics, School of Public Health, Tehran University of Medical Sciences (BA), Tehran, Iran; Digestive Disease Research Center, Digestive Diseases Research Institute, Tehran University of Medical Sciences (MA, AM, MB, FI, HP, AFS, AP, FK, AS, RM), Tehran, Iran; Digestive Oncology Research Center, Digestive Diseases Research Institute, Tehran University of Medical Sciences (MA, MB, HP, MHD, AFS, AP, AS, RM), Tehran, Iran; Department of Human Ecology, School of Public Health, Tehran University of Medical Sciences (MA), Tehran, Iran; Department of Epidemiology and Biostatistics, School of Public Health, Tehran University of Medical Sciences (MY), Tehran, Iran; Surveillance and Health Services Research, American Cancer Society (FI), Atlanta, GA; Institute for Translational Epidemiology and Tisch Cancer Institute, Icahn School of Medicine at Mount Sinai (PB), New York, NY; Division of Cardiovascular and Medical Sciences, Section of Gastroenterology, University of Glasgow (MHD), Glasgow, UK; Liver and Pancreatic-biliary Research Center, Digestive Diseases Research Institute, Tehran University of Medical Sciences (AP), Tehran, Iran; Division of Cancer Epidemiology and Genetics, National Cancer Institute (CCA, SMD), Bethesda, MD; International Agency for Research on Cancer, Genetic Epidemiology Group (PB), Lyon, France; and Department of Public Health Analysis, School of Community Health and Policy, Morgan State University (FK), Baltimore, MD.

## Abstract

Advances in medicine and health policy have resulted in growing of older population, with a concurrent rise in multimorbidity, particularly in Iran, as a country transitioning to a western lifestyle, and in which the percent of the population over the age of 60 years is increasing. This study aims to assess multimorbidity and the associated risk factors in Iran.

We used data from 50,045 participants (age 40–75 y) in the Golestan Cohort Study, including data on demographics, lifestyle habits, socioeconomic status, and anthropometric indices. Multimorbidity was defined as the presence of 2 or more out of 8 self-reported chronic conditions, including cardiovascular diseases, diabetes, chronic obstructive pulmonary disease, chronic kidney disease, liver disease, gastroesophageal reflux disease, tuberculosis, and cancer. Multivariate logistic regression models were used to examine the associations between multiple different factors and the risk factors.

Multimorbidity prevalence was 19.4%, with the most common chronic diseases being gastroesophageal reflux disease (76.7%), cardiovascular diseases (72.7%), diabetes (25.3%), and chronic obstructive pulmonary disease (21.9%). The odds of multimorbidity was 2.56-fold higher at the age of >60 years compared with that at <50 years (*P* < 0.001), and 2.11-fold higher in women than in men (*P* < 0.001). Other factors associated with higher risk of multimorbidity included non-Turkmen ethnicity, low education, unemployment, low socioeconomic status, physical inactivity, overweight, obesity, former smoking, opium and alcohol use, and poor oral health.

Apart from advanced age and female sex, the most important potentially modifiable lifestyle factors, including excess body weight and opium use, and opium user, are associated with multimorbidity. Policies aiming at controlling multimorbidity will require a multidimensional approach to reduce modifiable risk factors in the younger population in developing countries alongside adopting efficient strategies to improve life quality in the older population.

## INTRODUCTION

In most countries throughout the world, particularly in the developing countries, the proportion of the older people in the overall population is growing rapidly. This is a result of advances in medical science, technology, and health policy, resulting in a longer life expectancy and a decline in fertility rates.^1–3^ The senior population is expected to increase 3-fold in the next few decades in Iran. On the basis of estimates of the World Health Organization (WHO), by 2050, Iran, as a developing country, will have a greater proportion of older individuals (8.4%–29.4%) than the United States (20.1%–27%).^4–6^

In Iran, the aging population may be regarded as an indicator of successful population, and public health policies and socioeconomic development.^4^ The advances in health status of the Iranian population have transformed the most common diseases, from fatal acute diseases into survivable chronic conditions.^7^ The aging of the population will increase the absolute number of chronic conditions, causing people to suffer from multimorbidity, defined as the simultaneous occurrence of 2 or more chronic health disorders in the same person at one point in time.^8–10^ A systematic review of 21 studies showed that the estimated prevalence of multimorbidity varies widely in developed countries; it has been estimated that 1 in 4 adults in the developed countries experiences multimorbidity, and 50% of the older people have 2 or more chronic diseases.^11–13^ Data on multimorbidity from low and middle-income countries are sparse, and information about the health of older individuals in these regions is greatly needed. Studies predict that in 2050, 21.1% of world residents will be over 60 years old, and 80% of this group will live in low and middle-income countries.^14^

Multimorbidity is associated with higher mortality risk, functional disabilities, deterioration of quality of life, greater use of multiple medications with associated adverse effects, more frequent and longer hospitalization, and higher healthcare utilization and expenses.^9,12,15,16^ Because of the increasing importance of multimorbidity, we conducted a cross-sectional analysis of baseline data from the Golestan Cohort Study (GCS)—a large-scale study in a high incidence area of esophageal cancer in northern Iran^17^—to identify the prevalence and risk factors of multimorbidity in Iran as a middle-income country that may differ from those observed in developed countries.

## MATERIALS AND METHODS

### Study Population and Measurements

The study was carried out as a cross-sectional analysis of baseline information from the GCS. Details of the cohort enrollment are described elsewhere.^18^ Baseline data were collected during 2004 to 2008, and total enrollment included 50,045 adults aged 40 to 75 years residing in the Golestan Province, in northern Iran. Of the total participants, 49,946 (99.8%) were of Turkmen, Persian, Turkish, Sistani, Baluch, or Kurdish ethnicities; the other 99 enrollees were foreign nationals, who were excluded from this study.

Trained physician and nonphysician interviewers administered different parts of a lifestyle questionnaire to each participant in face-to-face interviews to collect information on age, sex, ethnicity, marital status, years of education, employment status, ownership of several appliances, physical activity, body mass index (BMI), smoking, opium and hookah (water pipe) use, alcohol consumption, and Decayed, Missing, Filled Teeth (DMFT) index. For each interviewee, a short physical examination was performed, and blood pressure, height, and weight were measured by trained general physicians.

In this mainly rural population, most of the physical activities performed by the participants were related to their jobs. Therefore, the physical activity was defined based on occupational activity, and coded as yes (heavy and intense activity) or no (all other participants). BMI was calculated using the WHO-recommended classification: underweight (BMI <18.5 kg/m^2^), normal (BMI 18.5–24.9 kg/m^2^), overweight (BMI 25–29.9 kg/m^2^), and obese (BMI >30 kg/m^2^). In this study, age at the time of interview was clustered as ≤49, 50 to 60, and >61 years. On the basis of the 2-step cluster analysis with the use of similarities of family asset, ethnicity, sex, employment status, age at starting the first job, size (surface area) and the status of house, age, and the status of house, we categorized the socioeconomic status (SES) of participants as low, middle, and high.^19,20^

Multimorbidity in this study refers to the presence of 2 or more chronic diseases.^8,10,15^ Similar to several comparable studies, the ascertainment of diseases was based on self-reports.^19,20^ The morbidities included cardiovascular diseases (CVDs), including hypertension, coronary heart disease and stroke, diabetes, chronic obstructive pulmonary disease (COPD), chronic kidney disease (CKD), liver disease, gastroesophageal reflux disease (GERD), tuberculosis, and cancers. We chose these diseases because they are frequently observed and have a major impact on health status, quality of life, and mortality in this population.^21–30^

The study was approved by the ethics committee of the Digestive Diseases Research Institute, Tehran University of Medical Sciences (OHRP-IRB-00001641). All participants signed an informed consent during original cohort study allowing investigators to use their anonymized data for further analysis.

### Statistical Analysis

We estimated the age and sex-standardized proportion of multimorbidity among participants by several sociodemographic and lifestyle factors. To evaluate the differences in distribution of multimorbidity by continuous or categorical factors, we used *t* tests, Mann–Whitney or chi-square tests, whenever appropriate. To examine the simultaneous effects of different factors and to calculate adjusted prevalence odds ratios (ORs) and 95% confidence intervals (CIs), we used multivariate logistic regression models; cluster effects were evaluated using the generalized estimating equation (GEE) method. All statistical analyses were performed using SPSS (Version 21.0, IBM Co. Chicago, IL). Two-sided *P* values below 0.05 were considered statistically significant.

## RESULTS

The study evaluated 49,946 individuals enrolled in the GCS. The mean age of participants was 52.1 ± 9 years, and the majority were women (57.6%), rural residents (76.8%), and of Turkmen ethnicity (74.6%). BMI was 25 kg/m^2^ or higher in 59.4% of the individuals, and 17% had used opium (Table 1).

**TABLE 1:**
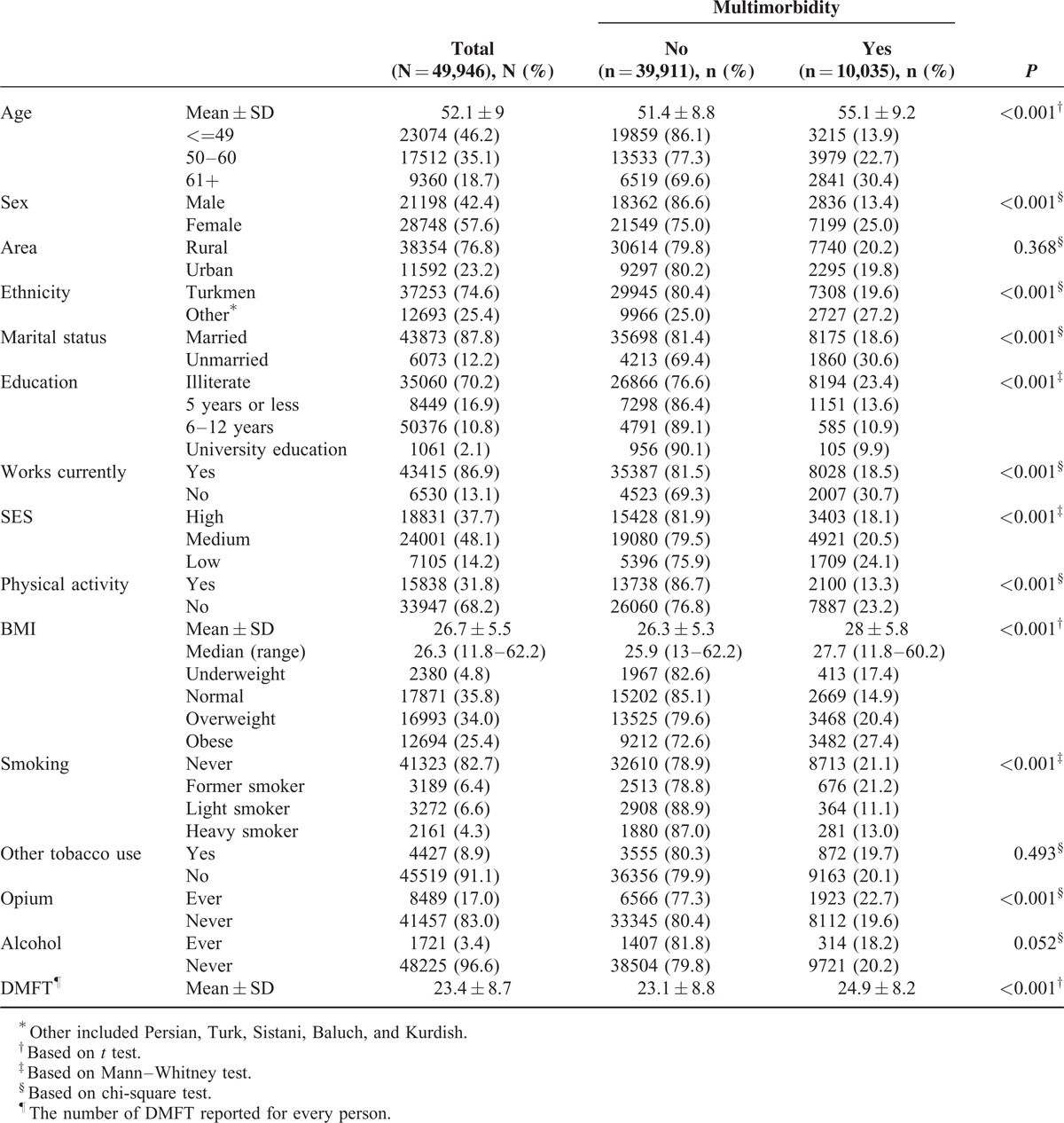
Distribution of Risk Factors in Patients With and Without Multimorbidity

In this study, the age and sex-standardized prevalence of multimorbidity was 19.4% (95% CI 19.1%–19.8%). The most common chronic diseases reported by those with multimorbidity were GERD (76.7%), CVD (72.7%), diabetes mellitus (25.3%), and COPD (21.9%). In multivariate models, older people, women, non-Turkmens, unemployed people, opium users, alcohol users, and those with low education, low physical activity, low SES, high BMI, and high DMFT (an indicator of poorer oral hygiene) were at a higher risk of multimorbidity (all *P* < 0.05) (Table 2).

**TABLE 2:**
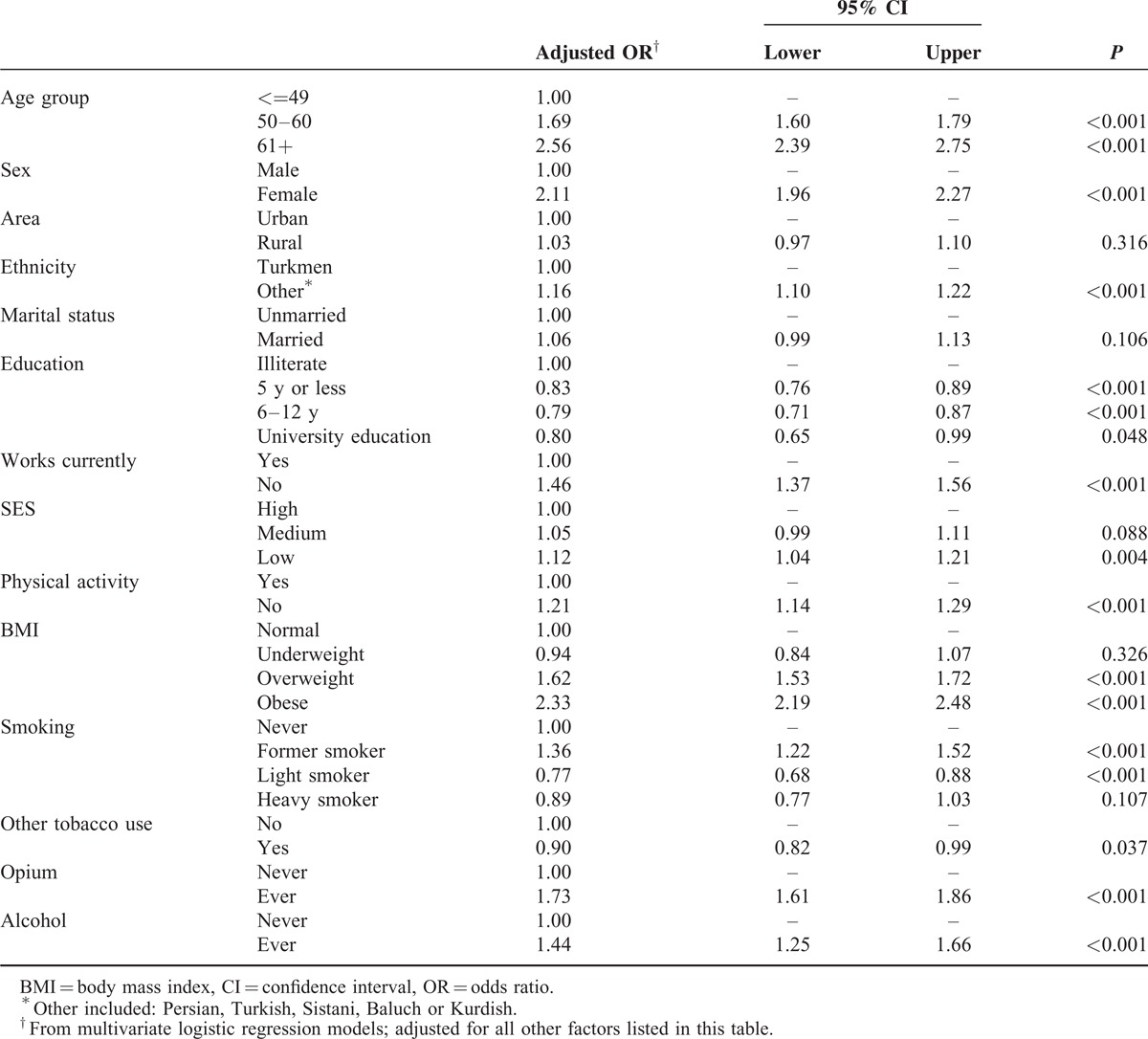
The Association Between Sociodemographic, Lifestyle Factors and Risk of Multimorbidity

Table 3 represent the prevalence of comorbidity in cases With multimorbidity. On the basis of these results, the most prevalent co-morbidity happened for CVDs and GERD (52%). The comorbidity of CVDs and diabetes was 18%.

**TABLE 3:**
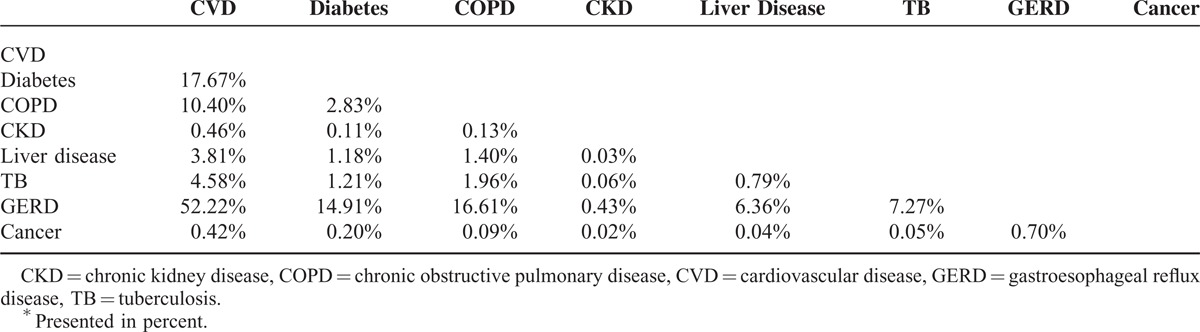
The Prevalence^∗^ of Comorbidity in Cases With Multimorbidity

**FIGURE 1:**
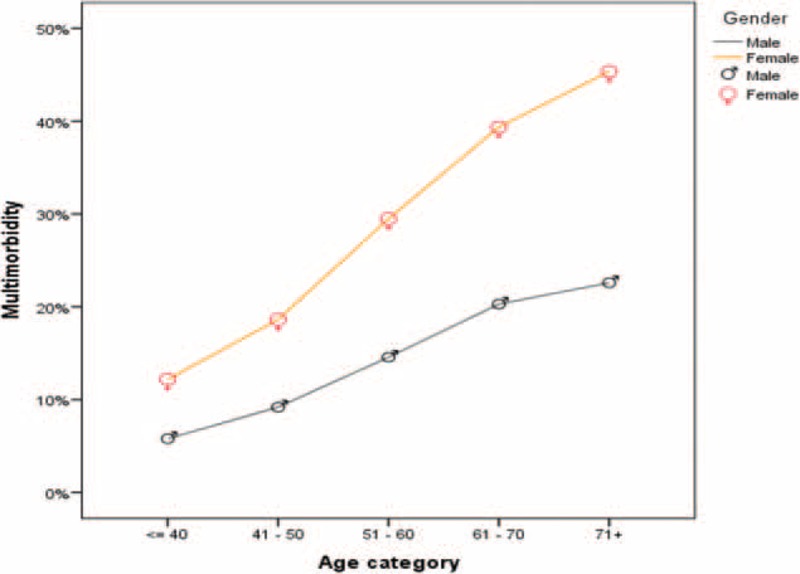
Prevalence of multimorbidity by age and sex.

Figure 1 shows the prevalence of multimorbidity by age and sex. In both sexes, multimorbidity increased with advancing age. In all age groups, the proportion of those with multimorbidity was higher in women than in men, and this difference increased with age.

The multivariate logistic regression models also showed that older age groups and women were at a higher risk of multimorbidity. The odds of multimorbidity was 2.56-fold higher in age groups above 60 years compared with those below 50 years (*P* < 0.001) and 2.11-fold higher in women than in men (*P* < 0.001). The odds of multimorbidity also increased significantly with higher BMI. For obese participants, the odds was 2.33-fold higher than those in the normal BMI group. Also, the risk of multimorbidity was 1.73-fold higher in opium users than in nonusers (*P* < 0.001).

**FIGURE 2:**
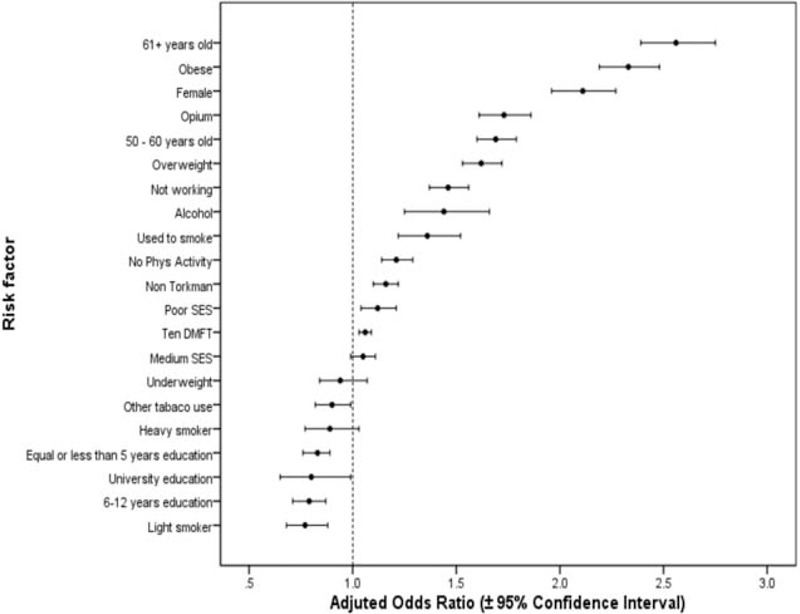
Odds ratios and 95% confidence intervals for the association between sociodemographic and lifestyle risk factors and multimorbidity^∗^ (^∗^in this graph, age <50 y, male sex, urban resident, Turkmen ethnicity, being unmarried, illiteracy, currently employment, high SES, physical activity, normal weight, never smoking, no other tobacco use, no opium use, and no alcohol use are considered as the reference group).

Figure 2 shows the adjusted ORs (95% CIs) for the association between a number of the above factors and multimorbidity in the multiple logistic regression models. The most important risk factors for multimorbidity were age above fifty years, obesity, female gender, opium use, and being overweight.

## DISCUSSION

In this large and unique population-based cohort study in Iran, a developing country in West Asia, the prevalence of self-reported multimorbidity was 19.4%. The most common self-reported chronic diseases in people with multimorbidity were GERD, CVD, diabetes, and COPD. Among the factors evaluated, age above fifty years, obesity, female gender, opium use, and being overweight were the most important risk factors for multimorbidity.

The prevalence of multimorbidity varies greatly across studies, largely because of differences in study populations and study methodologies. In particular, differences in the composition of studies in terms of age and other sociodemographic factors, definition of multimorbidity (including the number and types of diseases), and the applied statistics procedures seem to be responsible for the majority of this variation.^11,16,31-33^

The prevalence and risk factors of multimorbidity have usually been studied in high-income countries. To our knowledge, this is the first study of this subject conducted in Iran as a developing country. Our findings reveal that multimorbidity is a common phenomenon, with a prevalence of 19.4%. Similar to our study, Agborsangaya et al (2012) showed that the prevalence of multimorbidity was 19.0% among adults in the general population in Canada,^32^ and Phaswana-Mafuya et al (2008) reported a prevalence of 22.5% in individuals aged 50 years or above in South Africa.^34^ On the other hand, prevalence of multimorbidity in the general adult population reported by Loza et al (30%),^35^ Agborsangaya et al (31%)^36^ and Foguet-Boreu et al *(*46.8%)^8^ in developed countries were considerably higher than the prevalence that we found. Also, in a study by Nagel et al (2008), the prevalence of multimorbidity was 67.3% in the 50–75-year-old population in a prospective cohort study in Germany,^37^ which is considerably higher than our findings.

GERD is a common upper gastrointestinal disorder worldwide.^38^ The prevalence of GERD has been increasing in Iran in recent years.^39^ Our study showed that GERD as a possible risk factor of esophageal carcinoma was common in Golestan province, a high incidence area of esophageal carcinoma, which is in line with significant correlations of multimorbidity with lifestyle factors and SES. Lifestyle and sociodemographic factors such as BMI, intensity of physical activity, smoking, opium use, irregular dietary habits and low SES are all associated with GERD.^26,38^ CVD, diabetes mellitus, and COPD were also common among participants in the GCS. Many of the above lifestyle and sociodemographic factors are associated with these conditions. To some extent the coincidence of GERD and CVD may be due to the high frequency of both of these conditions in the area. Also, some evidences indicate that common inflammation pathway may be involved in the pathogenesis of GERD and following cardiovascular events.^40–42^

Our findings are consistent with previous studies, which have indicated that older age, obesity and female gender are associated with a higher prevalence of multimorbidity. The association between mulimorbidity and age is an expected finding, as most biological pathways leading to chronic diseases need a long time to affect individuals.^16,31,32,43,44^ Some studies showed that obesity was associated with at least a two-fold increased likelihood of having multimorbidity.^36^ This association may be explained by the fact that obese and overweight individuals are at increased risk of health problems and diseases such as coronary heart disease, certain cancers, diabetes, and pulmonary diseases.^44^

In our study population, opium consumption was common (17%) and significantly increased the risk of multimorbidity. Other studies showed that opium users had an increased risk of death from multiple causes, including circulatory diseases, cancer, asthma, tuberculosis, and COPD.^45,46^

Former smoking was a risk factor for multimorbidity in this study (OR of 1.36 compared with never smokers), while heavy smokers had a lower relative odd (0.89) for multimorbidity. Similar to our study, Chung et al (2015) showed that being past daily smoker was a significant risk factor for multimorbidity.^47^ This may be because people stop smoking after the symptoms of the related diseases appear.^48^

Regarding the indicators of SES in this study, after adjusting for other lifestyle and socio-demographic factors, the odds of multimorbidity was only 12% higher in the low SES group compared with the high SES group. This is contrary to some studies which found that multimorbidity was much more common in individuals of lower SES.^12,16^ The result in our study can be explained by the fact that the majority of the study participants were rural residents with relatively comparable SES. In addition, the number of people with a lower SES who knew about their nonsymptomatic medical conditions might be lower than those with a higher SES who might have a higher access to care.

During recent years, the population of Iranian adults above 60 years of age has increased to approximately 8%, similar to many developing countries.^49^ Demographic studies indicate that this population will rise to over 14% in the near future.^50^ A similar pattern is observed in other developing countries.^51^ Women have a longer life expectancy than men,^52^ and prevalence of multimorbidity increases with advancing age, especially in women, so the effects of population aging on multimorbidity should be more pronounced in women living in developing countries. GERD, CVD, diabetes and COPD are the most common diseases in this region, so it is important to plan for high burden of these diseases, which will be soon imposed on the healthcare system.

As a limitation, in interpreting our results, the differences in the quality of disease and death registration systems between developed and developing countries (i.e. Iran) should be taken into consideration. The lower rates of mortality and DALY (disability-adjusted life years) related to diabetes in eastern Mediterranean countries including Iran may be related to the lower quality of death and disease registration. Despite the almost similar diabetes mellitus prevalence in developing and western countries, the complications like renal disease were lower.^53^ This may be related to some extent to diabetes care in these countries. For instance Glycated hemoglobin testing as an index for diabetes surveillance was 72% in USA in comparison to 6.3% in Iran.^53^

The major strengths of this study are the use of data from a large, population-based cohort study running in a developing country including different races. As other strengths are the high participation rate and the availability of detailed data on sociodemographic and lifestyle factors. Also, biomarker studies, including comparison of opium intake data against codeine and morphine levels in urine samples, had shown the validity of the questionnaire for measuring exposure to some major risk factors.^54,55^ This study also has certain limitations, including its cross-sectional design and the potential for residual confounding and validation of self-report of diseases.

## CONCLUSION

Our study highlights the importance of multimorbidity in the Iranian population who experience an epidemiologic transition similar to many developing countries, and the importance of recognizing its risk and protective factors, especially in adults and the elderly. The findings of this study indicate that in addition to advanced age and female gender, some lifestyle factors, including being overweight, physical inactivity, and opium and tobacco use, are associated with multimorbidity. Policies aiming to control multimorbidity will require a multidimensional approach, especially in developing countries, to reduce modifiable risk factors in the younger population and adopt efficient strategies to improve quality of life in the adult and elderly population.
